# Using menopause status and 21-gene expression assay to inform chemotherapy benefit in node-positive breast cancer

**DOI:** 10.1007/s10549-022-06671-8

**Published:** 2022-07-10

**Authors:** Matthew G. Davey, Michael J. Kerin

**Affiliations:** The Lambe Institution of Translational Research, National University of Ireland, Galway, H91 YR71 Republic of Ireland

True Luminal A breast cancer represents an endocrine sensitive, surgically curable disease that does not mandate systemic chemotherapy as part of the management paradigm. This disease is reliably identified by a Recurrence Score (RS) < 25 using the OncotypeDX© 21-gene expression assay [1]. The recent RxPONDER trial update published by Kalinsky et al. builds on the seminal findings of the TAILORx trial [2], through highlighting the excellent oncological and survival outcomes now anticipated for patients diagnosed with early-stage Luminal A breast cancer, even in the setting of traditionally adverse prognostic factors, such as 1–3 positive nodes [3]. Regrettably, the trial’s conclusion focuses on the superficial finding that premenopausal patients who received combined chemoendocrine therapy (CET) have an invasive disease-free (iDFS) advantage of 4.9%, distant relapse-free survival of advantage 3.3%, which translated into just a 1.3% improvement in overall survival (OS) at 5-year follow-up. Therefore, the RxPONDER study reverberates the conclusion of the National Surgical Adjuvant Breast and Bowel Project (NSABP) B-20 trial, which recommended that all premenopausal breast cancer patients should be ‘candidates’ for systemic chemotherapy. While recent landmark trials have successfully opposed Fisher’s hypothesis [2, 3], this interpretation of the RxPONDER trial data exposes patients to the unnecessary toxicities of chemotherapy [1, 2]. The absolute benefit of CET becomes apparent when navigating the supplementary appendices accompanying the study, where the risk of disease recurrence in premenopausal patients treated with ET alone was 8.1% (76/826) versus 4.5% (37/829) for CET. The authors of RxPONDER then correctly conclude that the trial was not powered to accurately measure differences in subgroup divisions (including those based on menopause status). Surely, highlighting the large proportion of patients with RS < 25 who benefit from ET alone and the appropriate promotion of chemotherapy de-escalation for these young patients would serve as a more suitable conclusion to the RxPONDER trial?

Additionally, this conclusion from the RxPONDER trial lags considerably behind the de-escalation of surgery in early-stage breast cancer: There is a habitual tendency to prescribe untargeted chemotherapy in Luminal A breast cancer to improve survival, perhaps lessons could be learned from trials conducted in the advanced setting. In the recent MONALEESA-7 and PALOMA-3 trials, the addition of cyclin-dependent kinase 4 and 6 (CDK4/6) inhibitors to conventional endocrine agents enhanced survival for premenopausal women with metastatic Luminal A disease. Moreover, evidence from the SOFT and TEXT trials illustrate that adjuvant tamoxifen alone is appropriate for most premenopausal patients with early-stage disease, while absolute improvements of 10–15% observed in ‘high recurrence risk’ patients using ovarian suppression and exemestane versus tamoxifen alone. While it is unsurprising that CET prescription improves outcomes in premenopausal women, the oncological outcome observed from these targeted therapies far supersede the reported absolute benefit from CET described in the RxPONDER trial [2, 3]. While acknowledging the difficulties in addressing neoadjuvant response using endocrine treatment, translational neoadjuvant trials should evaluate these targeted approaches in premenopausal patients, like the strategy adopted to inform molecular substratification in the I-SPY2 trial.

The molecular insights from Paik et al. revealed the etiology of the small benefit in NSABP B-20 and prevented the perpetuation of the ongoing inappropriate therapeutic strategy [1]. Translation of this approach to a premenopausal cohort is required such that novel biomarker discovery, molecular subgroup analyses, and validation of targeted endocrine therapies capable of enhancing outcomes are investigated, in opposition to the current blunderbuss antiquated proposition of non-targeted CET with its large-scale toxicities. Such flaws are probably best exposed by the perceived inability of the RS to accurately clarify chemosensitivity in the premenopausal subgroup, which is contrary to several previous studies [1, 2]. Surely, opting for CET prescription in Luminal A breast cancer should be a matter of informed and personal patient choice.

Overall, the conclusion from this piece is quite simple: A treatment strategy that involves chemotherapy for patients with a RS < 25 risks the prolongation of a fallacy that opposes the scientific reasoning supporting personalized appropriate treatment strategies for breast cancer. A translational approach may help to minimize recurrence, maximize quality of life, and build on the novel insights gleaned from the molecular era. The next phase of neoadjuvant translational research trials must focus on deciphering the relatively small proportion of premenopausal patients with RS < 25 who are a recurrence risk and optimize the therapeutic strategy appropriately. Surely, we cannot prolong the suboptimal care of this generation or expose our prospective patients to untargeted toxic therapy which is neither justified nor effective for the majority.
